# Aspirin exerts high anti-cancer activity in *PIK3CA*-mutant colon cancer cells

**DOI:** 10.18632/oncotarget.20972

**Published:** 2017-09-18

**Authors:** Mancang Gu, Reiko Nishihara, Yang Chen, Wanwan Li, Yan Shi, Yohei Masugi, Tsuyoshi Hamada, Keisuke Kosumi, Li Liu, Annacarolina da Silva, Jonathan A. Nowak, Tyler Twombly, Chunxia Du, Hideo Koh, Wenbin Li, Jeffrey A. Meyerhardt, Brian M. Wolpin, Marios Giannakis, Andrew J. Aguirre, Adam J. Bass, David A. Drew, Andrew T. Chan, Charles S. Fuchs, Zhi Rong Qian, Shuji Ogino

**Affiliations:** ^1^ Department of Oncologic Pathology, Dana-Farber Cancer Institute and Harvard Medical School, Boston, MA, USA; ^2^ College of Pharmacy, Zhejiang Chinese Medical University, Hangzhou, P.R. China; ^3^ Department of Nutrition, Harvard T.H. Chan School of Public Health, Boston, MA, USA; ^4^ Department of Epidemiology, Harvard T.H. Chan School of Public Health, Boston, MA, USA; ^5^ Department of Biostatistics, Harvard T.H. Chan School of Public Health, Boston, MA, USA; ^6^ Program in MPE Molecular Pathological Epidemiology, Department of Pathology, Brigham and Women's Hospital and Harvard Medical School, Boston, MA, USA; ^7^ Medical Oncology Department 2, Chinese People's Liberation Army General Hospital, Beijing, P.R. China; ^8^ Department of Medical Oncology, Dana-Farber Cancer Institute and Harvard Medical School, Boston, MA, USA; ^9^ Broad Institute of MIT and Harvard, Cambridge, MA, USA; ^10^ Department of Medicine, Brigham and Women's Hospital and Harvard Medical School, Boston, MA, USA; ^11^ Clinical and Translational Epidemiology Unit, Massachusetts General Hospital and Harvard Medical School, Boston, MA, USA; ^12^ Division of Gastroenterology, Massachusetts General Hospital, Boston, MA, USA; ^13^ Yale Cancer Center, New Haven, CT, USA; ^14^ Department of Medicine, Yale School of Medicine, New Haven, CT, USA; ^15^ Smilow Cancer Hospital, New Haven, CT, USA

**Keywords:** anti-tumor effect, colorectal cancer, isogenic cell model, NSAID, PI3K

## Abstract

Evidence suggests that nonsteroidal anti-inflammatory drug aspirin (acetylsalicylic acid) may improve patient survival in *PIK3CA*-mutant colorectal carcinoma, but not in *PIK3CA*-wild-type carcinoma. However, whether aspirin directly influences the viability of *PIK3CA*-mutant colon cancer cells is poorly understood. We conducted *in vitro* experiments to test our hypothesis that the anti-proliferative activity of aspirin might be stronger for *PIK3CA*-mutant colon cancer cells than for *PIK3CA*-wild-type colon cancer cells. We measured the anti-proliferative effect of aspirin at physiologic concentrations in seven *PIK3CA*-mutant and six *PIK3CA*-wild-type human colon cancer cell lines. After exposure to aspirin, the apoptotic index and cell cycle phase of colon cancer cells were assessed. In addition, the effect of aspirin was examined in parental SW48 cells and SW48 cell clones with individual knock-in *PIK3CA* mutations of either c.3140A>G (p.H1047R) or c.1633G>A (p.E545K). Aspirin induced greater dose-dependent loss of cell viability in *PIK3CA*-mutant cells than in *PIK3CA*-wild-type cells after treatment for 48 and 72 hours. Aspirin treatment also led to higher proportions of apoptotic cells and G0/G1 phase arrest in *PIK3CA*-mutant cells than in *PIK3CA*-wild-type cells. Aspirin treatment of isogenic SW48 cells carrying a *PIK3CA* mutation, either c.3140A>G (p.H1047R) or c.1633G>A (p. E545K), resulted in a more significant loss of cell viability compared to wild-type controls. Our findings indicate that aspirin causes cell cycle arrest, induces apoptosis, and leads to loss of cell viability more profoundly in *PIK3CA*-mutated colon cancer cells than in *PIK3CA*-wild-type colon cancer cells. These findings support the use of aspirin to treat patients with *PIK3CA*-mutant colon cancer.

## INTRODUCTION

Colorectal cancer is the second leading cause of cancer-related deaths in the U.S. [1], and the prognosis for individuals with advanced colorectal cancer remains poor [2, 3]. Accumulating evidence indicates that colorectal cancer is a heterogeneous group of diseases, and that responsiveness to treatment varies from patient to patient [4–7]. This heterogeneity necessitates precision medicine approaches that take tumor molecular characteristics into account in order to predict an individual's response to a specific agent.

Aspirin (acetylsalicylic acid) is one of the most commonly used medications worldwide as an effective analgesic, antipyretic and cardiovascular prophylactic agent. Several observational studies and randomized controlled trials have shown that regular use of aspirin after a diagnosis of colorectal cancer is associated with a superior clinical outcome [8–14]. Randomized trials of aspirin for prevention of cardiovascular and cerebrovascular events also suggest that regular aspirin use has the potential to reduce colorectal cancer incidence and mortality [15, 16]. Recently, the U.S. Preventive Services Task Force (USPSTF) recommended the use of aspirin for prevention of colorectal cancer in individuals at a 10% increased risk for cardiovascular disease compared with the general population [17], while cautioning against the potential harms associated with regular aspirin use including gastrointestinal pain and bleeding [18]. Therefore, it is of particular interest to identify subgroups of individuals who are most likely to benefit from aspirin-based therapeutic strategies through the development of informative tumor biomarkers [14].

In line with this precision medicine approach, multiple observational studies of colorectal cancer patients have indicated that the beneficial effects of aspirin may be stronger for *PIK3CA*-mutant colorectal cancer than for *PIK3CA*-wild-type tumors [12, 13, 19], though other studies have shown alternate findings [20, 21]. *PIK3CA* mutations are observed in approximately 15% to 20% of human colorectal carcinomas, and are associated with proximal tumor location [22–25] and *KRAS* mutations [26–28]. *PIK3CA* mutations can activate multiple oncogenic pathways, including the phosphatidylinositol-4,5-bisphosphonate 3-kinase (PI3K)/*AKT*/*MTOR*, *WNT*/*CTNNB1*, and *NFKB* signaling pathways [29–31]. Studies have shown that PI3K upregulation also enhances prostaglandin-endoperoxide synthase-2 (*PTGS2*, also known as cyclooxygenase-2) activity and prostaglandin E_2_ synthesis [32], which promotes cancer cell proliferation and inhibits apoptosis [33, 34]. Therefore, we hypothesized that the anti-cancer effects of aspirin might be stronger for *PIK3CA*-mutant colon carcinoma cell lines than for *PIK3CA*-wild-type colon carcinoma cell lines.

To test this hypothesis, we assessed the effect of aspirin treatment on cell cycle arrest and apoptosis using 13 colon cancer cell lines with known *PIK3CA* mutation status and isogenic colon cancer cells with knock-in *PIK3CA*-activating mutations. Additionally, our study identified cell models that may serve as ideal *in vitro* models to further clarify the molecular mechanisms of aspirin's anti-tumor effects in *PIK3CA*-mutant colorectal cancer.

## RESULTS

### Strong cell viability reduction by aspirin in *PIK3CA*-mutant colon cancer cells

**Figure 1:**
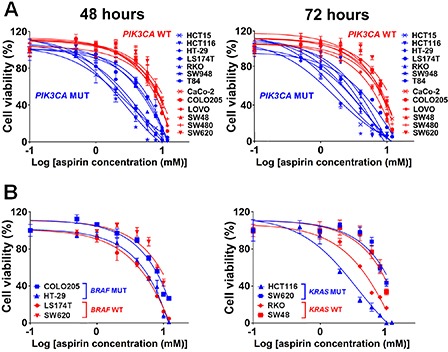
Aspirin causes decreased cell viability in *PIK3CA*-mutant human colon cancer cells (**A**) Dose-response curves of *PIK3CA*-mutant (blue lines) or *PIK3CA*-wild-type (red lines) human colon cancer cells after treatment with increasing concentrations of aspirin (0, 0.5, 1, 2, 4, 6, 8, 10 and 12 mM) for 48 and 72 hours. (**B**) Dose-response curves of human colon cancer cells *with BRAF*-mutant (blue lines) or *BRAF*-wild-type (red lines) as well as *KRAS*-mutant (blue lines) or *KRAS*-wild-type (red lines) after treatment with increasing concentrations of aspirin (0, 0.5, 1, 2, 4, 6, 8, 10 and 12 mM) for 48 hours. Percent cell viability is relative to that of DMSO-treated control cells. The data shown represent mean ± standard deviation of three replicates. DMSO, dimethyl sulfoxide; WT, wild-type; MUT, mutation.

To test the hypothesis that aspirin's anti-cancer effects might differ by *PIK3CA* mutation status, we investigated the anti-proliferative activity of aspirin at physiologically attainable concentrations in seven *PIK3CA*-mutant and six *PIK3CA*-wild-type colon cancer cell lines (Table 1) using the 3-(4,5-dimethylthiazol-2-yl)-5-(3-carboxymethoxyphenyl)-2-(4-sulfophenyl)-2H- tetrazolium inner salt (MTS) assay. Initially, we examined the effect of a variety of aspirin doses (0, 0.5, 1, 2, 4, 6, 8, 10, and 12 mM) on cell proliferation after exposure to aspirin for 12 to 96 hours in two *PIK3CA*-mutant (HCT15 and HCT116) and *PIK3CA*-wild-type (SW480 and SW620) colon cancer cell lines. We found that the most prominent effects of aspirin on proliferation occurred between 48 and 72 hours of exposure. Therefore, all subsequent experiments were performed after treatment with aspirin for 48 and 72 hours. The dose-response curves of *PIK3CA*-mutant and *PIK3CA*-wild-type colon cancer cell lines were generated (Figure 1) and the half maximal inhibitory concentration (IC_50_) values of each *PIK3CA* subgroup were compared (Supplementary Figure 1A). *PIK3CA*-mutant colon cancer cell lines were considerably more sensitive to the anti-proliferative effects of aspirin than *PIK3CA*-wild-type cells (Figure 1A). Aspirin did not show differential effects according to *BRAF* or *KRAS* mutation status (Figure 1B). Next, in order to validate that *PIK3CA*-mutant colon cancer cell lines were specifically sensitized to aspirin but not to common DNA damaging chemotherapeutic agents, we investigated the IC_50_ values of 5-fluorouracil and cisplatin in our thirteen colon cancer lines from a compound sensitivity database of the Cancer Cell Line Encyclopedia (CCLE) [35]. However, no significant differences were found between *PIK3CA*-mutant and *PIK3CA*-wild-type colon cancer cell lines (Supplementary Figure 1B).

**Table 1:** Mutational status of *PIK3CA*, *BRAF*, and *KRAS* in colon cancer cell lines used in the current study

Cell line	Mutation status^*^		
	*PIK3CA*	*BRAF*	*KRAS*
HCT15	c.1633G>A (p.E545K),c.1645G>A (p.D549N)	WT	c.38G>A (p.G13D)
HCT116	c.3140A>G (p.H1047R)	WT	c.38G>A (p.G13D)
HT-29	c.1345C>A (p.P449T)	c.1799T>A (p.V600E)	WT
LS174T	c.3140A>G (p.H1047R)	WT	c.35G>A (p.G12D)
RKO	c.3140A>G (p.H1047R)	c.1799T>A (p.V600E)	WT
SW948	c.1624G>A (p.E542K)	WT	c.182A>T (p.Q61L)
T84	c.1624G>A (p.E542K)	WT	c.38G>A (p.G13D)
CaCo-2	WT	WT	WT
COLO205	WT	c.1799T>A (p.V600E)	WT
LOVO	WT	WT	c.38G>A (p.G13D), c.41C>T (p.A14V)
SW48	WT	WT	WT
SW480	WT	WT	c.35G>T (p.G12V)
SW620	WT	WT	c.35G>T (p.G12V)

^*^Mutation according to the American Type Culture Collection. WT, wild-type.

### Aspirin induces apoptosis and G_0_/G_1_ cell cycle arrest in *PIK3CA*-mutant cells

**Figure 2:**
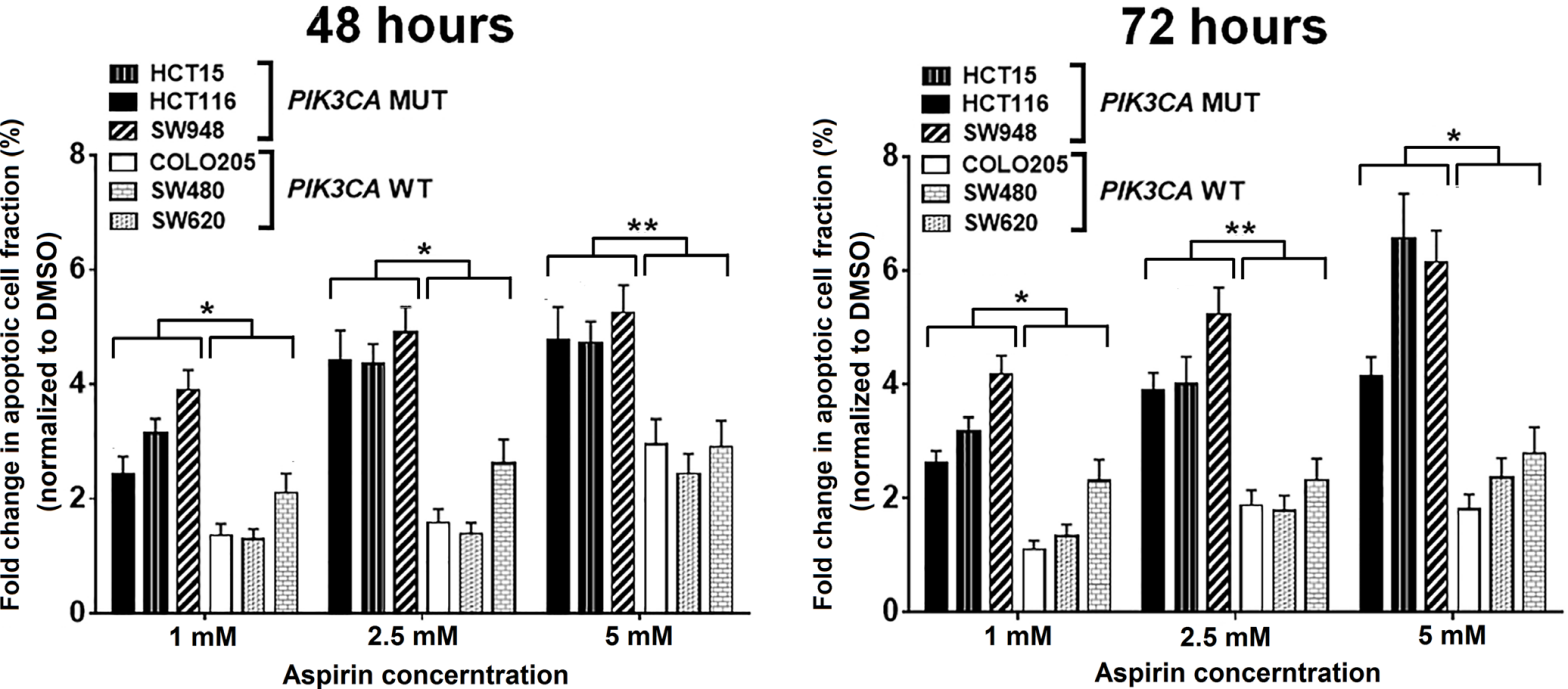
Aspirin treatment results in more apoptosis in *PIK3CA*-mutant human colon cancer cell lines *PIK3CA*-mutant human colon cancer cell lines (HCT15, HCT116, and SW948) and *PIK3CA*-wild-type colorectal cancer cell lines (COLO205, SW480, and SW620) were incubated with aspirin (1, 2.5, and 5 mM) or DMSO for 48 and 72 hours. Apoptosis analysis was performed by flow cytometry with Annexin V-FITC/PI staining. The percentage of Annexin V-FITC/PI positive cells after aspirin treatment at each dose and time point for all cell lines were normalized by DMSO group and compared according *PIK3CA* status. Student's *t*-test was performed to determine significance. The data shown represent mean ± standard deviation of three replicates. ^*^P value < 0.05. ^**^P value < 0.01. DMSO, dimethyl sulfoxide; MUT, mutation; WT, wild-type.

**Figure 3:**
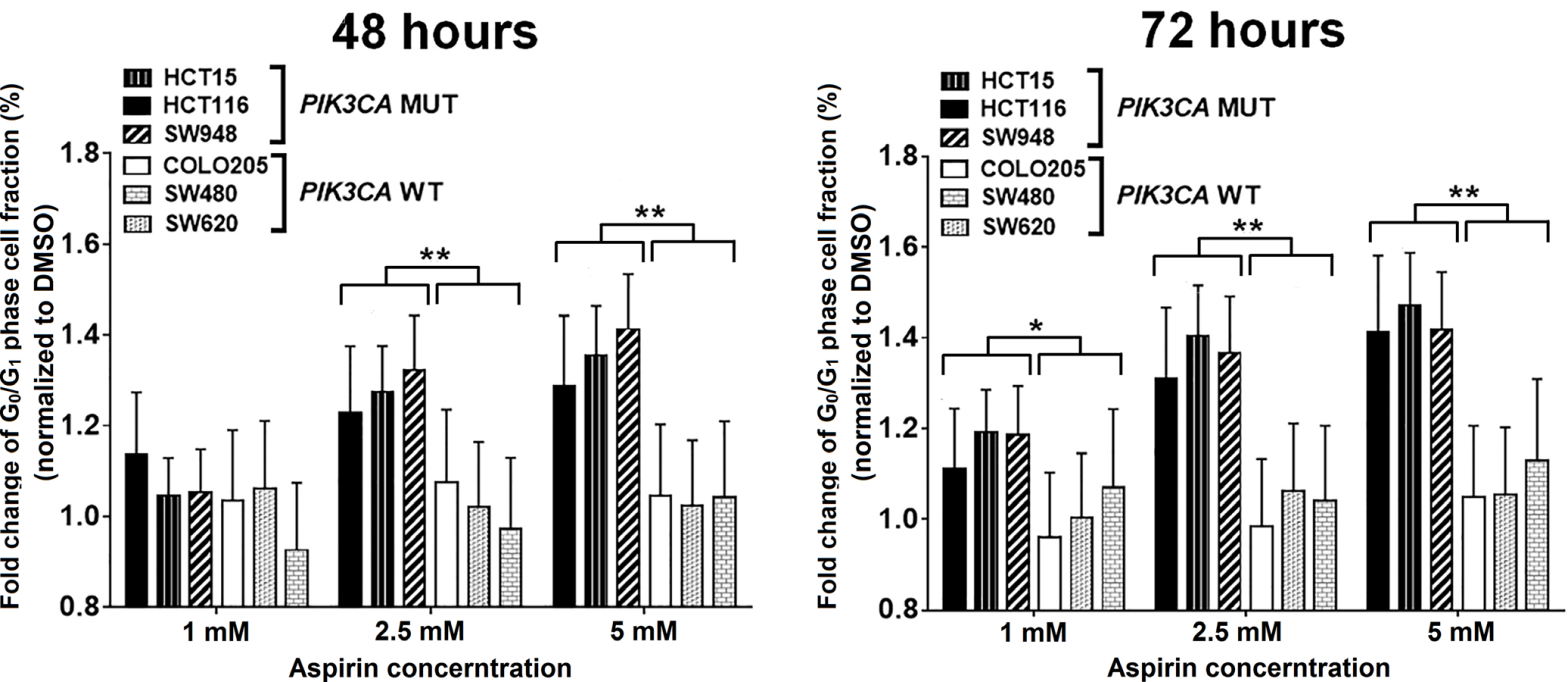
Aspirin leads to a higher proportion of cells in G_0_/G_1_ phase arrest in *PIK3CA*-mutant human colon cancer cell lines *PIK3CA*-mutant human colon cancer cell lines (HCT116, HCT15, and SW948) and *PIK3CA*-wild-type colorectal cancer cell lines (COLO205, SW620, and SW480) were incubated with aspirin (0, 1, 2.5, and 5 mM) for 48 and 72 hours. Cell cycle analysis was performed by flow cytometry with PI staining. The percentage of G_0_/G_1_ phase cells of each aspirin treatment point was normalized by DMSO group and was compared according *PIK3CA* status. Student's *t*-test was performed to determine significance. The data shown represent mean ± standard deviation of three replicates. ^*^P value < 0.05. ^**^P value < 0.01. DMSO, dimethyl sulfoxide; MUT, mutation; WT, wild-type.

We evaluated the effects of aspirin on induction of apoptosis and cell cycle distribution in three *PIK3CA*-mutant cell lines (HCT15, HCT116, and SW948) that were particularly sensitive to aspirin exposure in the MTS assay and three *PIK3CA*-wild-type colon cancer cell lines (COLO205, SW480, and SW620) that were resistant to aspirin exposure. Cells were treated with 0, 1, 2.5, and 5 mM aspirin for 48 and 72 hours after plating. Aspirin treatment led to a more profound induction of apoptosis in colon cancer cells harboring a *PIK3CA* mutation than in those with wild-type *PIK3CA* (Figure 2). We next assessed the percentage of cells in each phase of the cell cycle within these *PIK3CA* mutant and wild-type colon cancer cells (Figure 3). All *PIK3CA*-mutant cell lines underwent a dose-dependent increase in G_0_/G_1_ cell cycle arrest after aspirin exposure for 48 and 72 hours. This effect was not observed in *PIK3CA*-wild-type colon cancer cell lines.

### Mutations in *PIK3CA* oncogene sensitize colon cancer cells to aspirin

**Figure 4:**
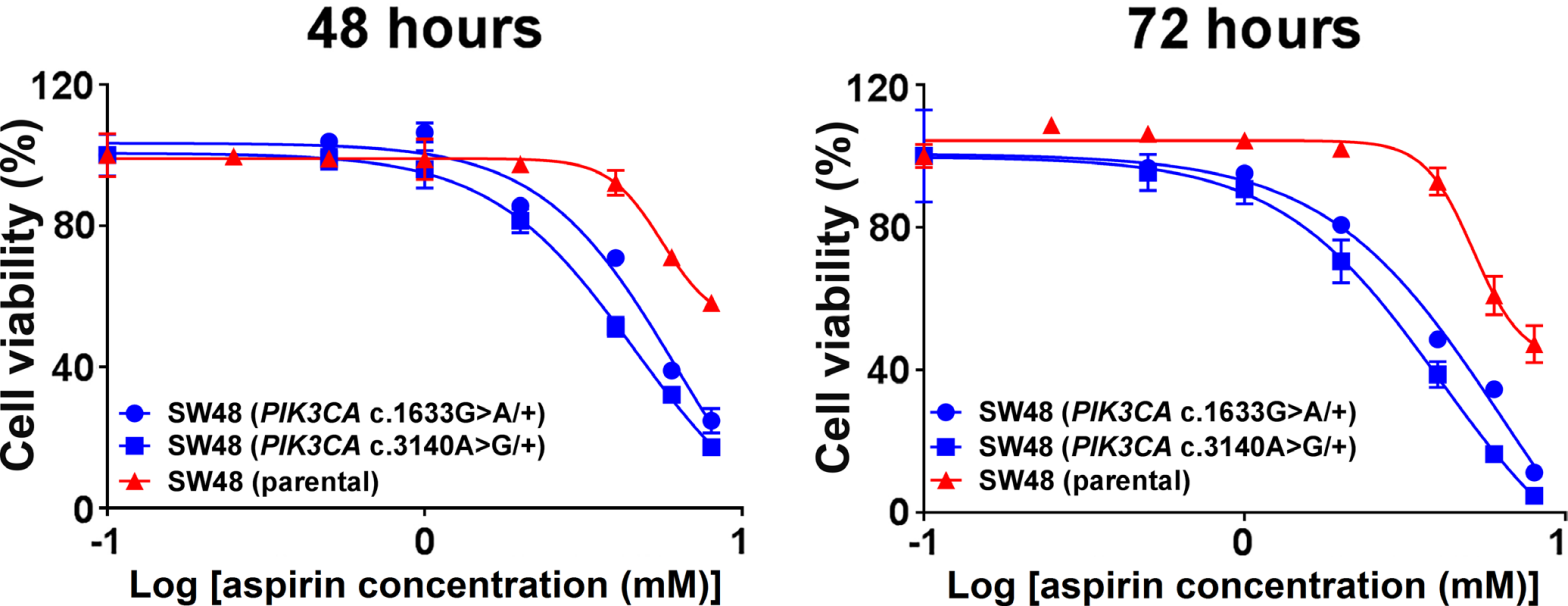
Knock-in of *PIK3CA* mutations sensitizes colon cancer cells to aspirin Dose-response curves of parental SW48 cells (red) and individual knock-in of *PIK3CA*-activating mutations at either alleles c.3140A>G (p.H1047R) or c.1633G>A (p.E545K) of SW48 cells (blue) were treated with aspirin (0, 0.5, 1, 2, 4, 6 and 8 mM) for 48 and 72 hours. Percent cell viability is relative to that of DMSO-treated control cells. The data shown represent mean ± standard deviation of three replicates. DMSO, dimethyl sulfoxide; MUT, mutation; WT, wild-type.

The *PIK3CA* c.3140A>G (p.H1047R) and c.1633G>A (p.E545K) somatic mutations are commonly found in colorectal carcinoma, and were present in the colon cancer cells (HCT15 and HCT116, respectively) included in the published studies [36, 37]. In order to further confirm our findings and ensure that the observed *PIK3CA*-mutant-specific effect was not confounded by the genomic backgrounds of the selected cell lines, we investigated whether activation of PI3K via knock-in of the c.3140A>G (p.H1047R) or c.1633G>A (p.E545K) mutation could sensitize a *PIK3CA* wild-type colon cancer cell line to aspirin treatment. Two isogenic cell lines were derived from parental SW48 cells, each of which carried constitutively active mutant alleles with mutations at either c.3140A>G [SW48 (*PIK3CA* c.3140A>G/+)] or c.1633G>A [SW48 (*PIK3CA* c.1633G>A/+)]. The IC_50_ values of each group showed that aspirin treatment of isogenic SW48 cells carrying either *PIK3CA* mutation resulted in a statistically significant loss of cell viability of up to 47% relative to parental SW48 cells (*P* = 0.031) (Figure 4).

## DISCUSSION

We conducted this study to provide *in vitro* experimental evidence supporting the recent molecular pathological epidemiology studies that suggest tumor *PIK3CA* mutation status as a biomarker to predict benefits from aspirin therapy for colorectal cancer [9, 12, 13, 21, 38]. In this study, we have shown that physiologically attainable concentrations of aspirin can exert stronger anti-cancer effects on *PIK3CA*-mutant colon cancer cells relative to *PIK3CA*-wild-type colon cancer cells.

Our results are consistent with a recent report by Zumwalt *et al* [39] that clarified the relationship of *PIK3CA* mutations in colorectal cancer cells and aspirin-induced chemoprevention *in vitro* and *in vivo*. In that study, eight colon cancer cell lines and engineered HCT116 cells with *PIK3CA* kinase domain mutant allele knockout were used to assess the effects of aspirin on cell proliferation and cell-cycle distribution. Our current study further investigated 13 commonly used colon cancer cell lines and two isogenic cell lines with heterozygous knock-in of either of *PIK3CA* mutations c.3140A>G (p.H1047R) and c.1633G>A (p.E545K), both of which are commonly observed in colorectal carcinoma. In addition, aspirin treatment of breast cancer cells carrying mutations in *PIK3CA* at either exon 9 (c.1633G>A) or exon 20 (c.3140A>G) also resulted in a significant decrease of cell viability [40].

Although multiple lines of evidence indicate that aspirin use after colorectal cancer diagnosis is beneficial, the toxicities of therapy, particularly in older patients, have limited its use in routine clinical practice [14, 19, 41]. In 2016, the USPSTF gave a “B” recommendation (high certainty that the net benefit is moderate or moderate certainty that the net benefit is moderate or substantial) for routine aspirin use for colorectal cancer prophylaxis in U.S. adults between the ages of 50 and 59 with a greater than 10% 10-year risk of cardiovascular events [17]. The USPSTF recommendations also highlight the need to clarify the mechanisms by which aspirin prevents development of colorectal cancer [18]. There are currently several clinical trials underway (including ADD-ASPIRIN [42], ASCOLT [43], and ASPIRED [44]) that are attempting to clarify the association between aspirin use and survival after diagnosis of colorectal cancer, as well as distinguishing the subgroups of individuals for whom the benefits outweigh the harm. Thus, it is essential to confirm the efficacy of biomarkers used to predict benefits from aspirin therapy through multifaceted approaches, including clinical trials and *in vitro* or *in vivo* experimental models [14]. The present study serves this purpose by improving our understanding of the use of tumor *PIK3CA* mutation status for prediction of patient response to aspirin therapy.

*PIK3CA* mutations (or *PIK3CA* amplifications [45]) may result in constitutive activation of PI3K and the downstream *AKT* pathway, enhancing *PTGS2* activity and prostaglandin E_2_ synthesis and leading to inhibition of apoptosis in colorectal cancer cells [11]. Recent *in vitro* and *in vivo* studies have suggested that aspirin may suppress cancer cell growth and induce apoptosis through activation of protein kinase A (*PRKA*, also referred to as AMPK), inhibition of *MTOR* downstream signaling, and inhibition of PI3K-induced prostaglandin E_2_ synthesis [46–48]. The present study was designed to detect the potency of aspirin therapy in colon cancer cell lines according to major somatic driver mutations such as *PIK3CA, BRAF*, and *KRAS* mutations. The results demonstrate that aspirin may selectively cause G_0_/G_1_ cell cycle arrest, induce apoptosis, and inhibit cell growth in human colon cancer cells with *PIK3CA* mutations *in vitro*. Our findings in the apoptotic and cell cycle assays are consistent with the data on the previous *in vitro* studies [39, 40, 47, 48]. The reduced viability of *PIK3CA*-mutant colon cancer cells was also confirmed in colon cancer cells containing the heterozygous *PIK3CA* knock-in mutations c.3140A>G (p.H1047R) and c.1633G>A (p.E545K). Previous retrospective analyses investigating aspirin use in colon cancers according to *PIK3CA* mutation status did not specify which *PIK3CA* mutations were included [10, 12]. The present study is the first to show that the isogenic conversion of a *PIK3CA*-wild-type colon cancer cell into a *PIK3CA*-mutant cell line is sufficient to promote sensitization to aspirin. *PIK3CA* c.3140A>G (p.H1047R) and c.1633G>A (p.E545K) mutations are common in colorectal carcinomas. Heterozygous knock-in c.3140A>G and c.1633G>A mutations in the *PIK3CA* gene were found to activate multiple oncogenic pathways and promote cell growth and invasion *in vitro* for human breast cancer and colon cancer cell lines [37, 49]. Notably, breast cancer cells carrying a c.3140A>G (p.H1047R) mutation at exon 20 were less sensitive to aspirin than those carrying a c.1633G>A (p.E545K) mutation at exon 9 [40]. This phenomenon may be attributed to differences in the aberrant activation of signaling pathways. However, few, if any, differences in phenotype were observed according to *PIK3CA* mutation variants.

The relationship between somatic or germline genetic variants and the chemopreventive use of aspirin is not limited to patients with *PIK3CA*-mutant colorectal cancer. Lizaka and colleagues utilized a genome-wide complementary DNA microarray containing 23,040 genes to analyze the time-dependent alteration of gene expression in response to two nonsteroidal anti-inflammatory drugs (NSAIDs), sulindac and aspirin, in SNU-C4, SW480, and SW948 colon cancer cell lines [50]. Interestingly, they found that *PIK3C2A*, which belongs to the *PIK3* family, was down-regulated only in aspirin-sensitive cells. We previously performed a genome-wide analysis of the interactions between single nucleotide polymorphisms (SNPs) and aspirin use in relation to colon cancer risk, which identified two SNPs on chromosomes 12 and 15 that differentially correlate with aspirin and NSAID effects on colon cancer prevention [51]. One, on chromosome 12, is located upstream from *PIK3C2G*, which belongs to the *PIK3* family. Combined, these findings further support the use of *PIK3CA* mutation status as a biomarker for precision aspirin chemoprevention and adjuvant therapy strategies for colon cancer.

Our current study has limitations. As our colon cancer cell panel did not include all possible activating alterations in *PIK3CA*, we were unable to elucidate the potency of aspirin treatment on colon cancer cells with *PIK3CA* alterations other than the ones examined. In addition, our current cancer cell panel was limited to immortalized colon cancer cell lines due to the lack of reasonable *PIK3CA*-mutant and *PIK3CA*-wild-type rectal cancer cell line pairs in the American Type Culture Collection (ATCC). Taking into account the potential heterogeneity of tumor behavior between colon and rectal carcinomas, anti-cancer effects of aspirin according to *PIK3CA* mutation status in rectal cancer cells and human primary cells should be examined in future studies. In the present study, the aspirin concentrations utilized in apoptosis and cell cycle analyses are relatively high (as high as 5 mM) compared to typical plasma concentrations of salicylate (0.19 to 0.63 mM), a major metabolite derived from aspirin. Thus, the doses may not be attainable through administration of standard doses (81 or 325 mg) of aspirin. However, the dose-dependent nature of the response to aspirin treatment suggests that some effects may be experienced *in vivo* even at low doses. Furthermore, Turturro *et al*. reported that the serum levels of aspirin could safely reach concentrations of up to 10 mM [40], indicating that the 5 mM aspirin dose is physiologically achievable in humans. Our *in vitro* models did not take into account the critical role of the tumor stromal microenvironment. Accumulating evidence indicates that regular aspirin use may synergize with other immunomodulatory pathways including the immune checkpoint blockade in colorectal cancer [52–56]. Further investigations are warranted to analyze interactions of multiple cell types that exist in the tumor microenvironment, and to examine the dual blockade of the *PTGS2* and immunosuppressive pathways. Another drawback is that our current study did not utilize functional assays to investigate potential molecular mechanisms through which aspirin may exert stronger anti-cancer effects on *PIK3CA*-mutant colon cancer cells than *PIK3CA*-wild-type cells. Nonetheless, our study hypothesis was based on human clinical studies [8–13, 57, 58]. It is important to perform functional studies to explore potential mechanisms underlying our findings in the future. We point out an important synergism of experimental research and molecular pathological epidemiology (MPE) research [59–61] as replicating biological complexities of human tumors in experimental models is a substantial challenge. In addition, it is of particular interest to investigate modifying effects of other endogenous and exogenous factors (such as diet, lifestyle, microbiota, germline genetics, and immunity) on medications (such as aspirin) in relation to tumor molecular characteristics [4, 6, 7] in integrative pharmaco-MPE research [62, 63] in the future.

In summary, our findings provide valuable *in vitro* data to further support the use of aspirin in patients with *PIK3CA*-mutant colorectal cancer. Further investigations into the precise molecular mechanisms associated with aspirin treatment of cancers with mutant *PIK3CA* are warranted.

## MATERIALS AND METHODS

### Cell lines and culture

A panel of 13 human colon cancer cell lines (CaCo-2, COLO205, HCT15, HCT116, HT-29, LOVO, LS174T, RKO, SW48, SW480, SW620, SW948, and T84) was obtained from the American Type Culture Collection (ATCC, Manassas, VA, USA). The mutational gene backgrounds of the cell lines were described previously [35, 64]. We used X-MAN™ isogenic cell lines, SW48 (*PIK3CA* c.1633G>A (p.E545K); Catalog # HD103-001) and SW48 (*PIK3CA* c.3140A>G (p.H1047R); Catalog # HD103-005) heterozygous knock-in of *PIK3CA* activating mutations, as well as SW48 parental cells (Catalog # HD PAR-006) (Horizon Discovery, Cambridge, MA, USA). The parental cell line SW48 was used as a control. All cells were cultured in RPMI-1640 (Gibco, Grand Island, NY, USA) supplemented with 10% fetal bovine serum (Gibco) and 100 U/ml of penicillin-streptomycin in a 5% CO_2_ and 95% air humidified atmosphere at 37°C. All colon cancer cell lines used in this study passed the STR (Short Tandem Repeat) authentication by manufacturers.

### Cell proliferation MTS assay

Cells were seeded in 96-well microplates at a density of 4×10^4^ cells/ml and cultured overnight; they were treated with a range of aspirin (Sigma-Aldrich, St. Louis, MO, USA) doses (0, 0.5, 1, 2, 4, 6, 8, 10 and 12 mM) in dimethyl sulfoxide (DMSO). These doses are representative of physiologically attainable plasma concentrations as previously reported [40]. Control cells received DMSO only. Cell proliferation was assessed after 48 hours and 72 hours from initial drug exposure using the MTS assay (Promega, Madison, WI, USA) according to the manufacturer's instructions. Cell proliferation is directly proportional to the absorbance at 490 nm by a formazan product that is bio-reduced from MTS in living cells. All experiments were carried out in hexaplicate and were repeated at least three times independently.

### Apoptosis and cell cycle analysis

The apoptosis levels and cell cycle phase of colorectal cancer cells after aspirin exposure were assessed as previously described [65, 66]. In brief, for apoptosis detection, trypsinized cells were collected gently and stained with the Annexin V-fluorescein isothiocyanate (FITC)/propidium iodide (PI) Apoptosis Detection kit (BD Biosciences, San Jose, CA, USA) according to the manufacturer's protocol. Cell cycle analysis was performed using PI staining. Cells fixed with 80% ethanol overnight at 4°C were resuspended in phosphate-buffered saline supplemented with 0.1% Triton X-100 (Sigma-Aldrich, St Louis, MO, USA), 25 mg/ml PI (BD Biosciences) and 0.2 mg/ml RNase A (Sigma-Aldrich), then incubated for 30 minutes at room temperature in the dark before analysis. Apoptosis and cell cycle phase were measured on a FACS Ariall cytometer (BD Biosciences), and analysis was performed using FlowJo software (FLOWJO, Ashland, OR, USA).

### Statistical analysis

Graphpad Prism 6.0 software (Graphpad Software, San Diego, CA, USA) was used to describe dose-response curves and perform statistical analysis. All data were represented for at least three independent experiments. The Student's *t*-test was used to compare continuous measurements in two groups. A *P* value less than 0.05 was considered statistically significant.

## SUPPLEMENTARY MATERIALS FIGURE



## Abbreviations

DMSO: dimethyl sulfoxide
IC_50_: half maximal inhibitory concentration
MTS: 3-(4,5-dimethylthiazol-2-yl)-5-(3-carboxymethoxyphenyl)-2-(4-sulfophenyl)-2H-tetrazolium inner salt
NSAID: nonsteroidal anti-inflammatory drug
PI: propidium iodide
PI3K: phosphatidylinositol-4,5-bisphosphonate 3-kinase
SNP: single nucleotide polymorphism
USPSTF: the U.S. Preventive Services Task Force
