# Supplementary material for: Aspirin exerts high anti-cancer activity in PIK3CA-mutant colon cancer cells
Source: Oncotarget. 2017 Sep 18;8(50):87379–89. doi: 10.18632/oncotarget.20972 (PMC5675640; doi:10.18632/oncotarget.20972)
Supplement: Supplementary file 1 [file oncotarget-08-87379-s001.pdf]

Aspirin exerts high anti-cancer activity in *PIK3CA*-mutant colon cancer cells

SUPPLEMENTARY MATERIALS

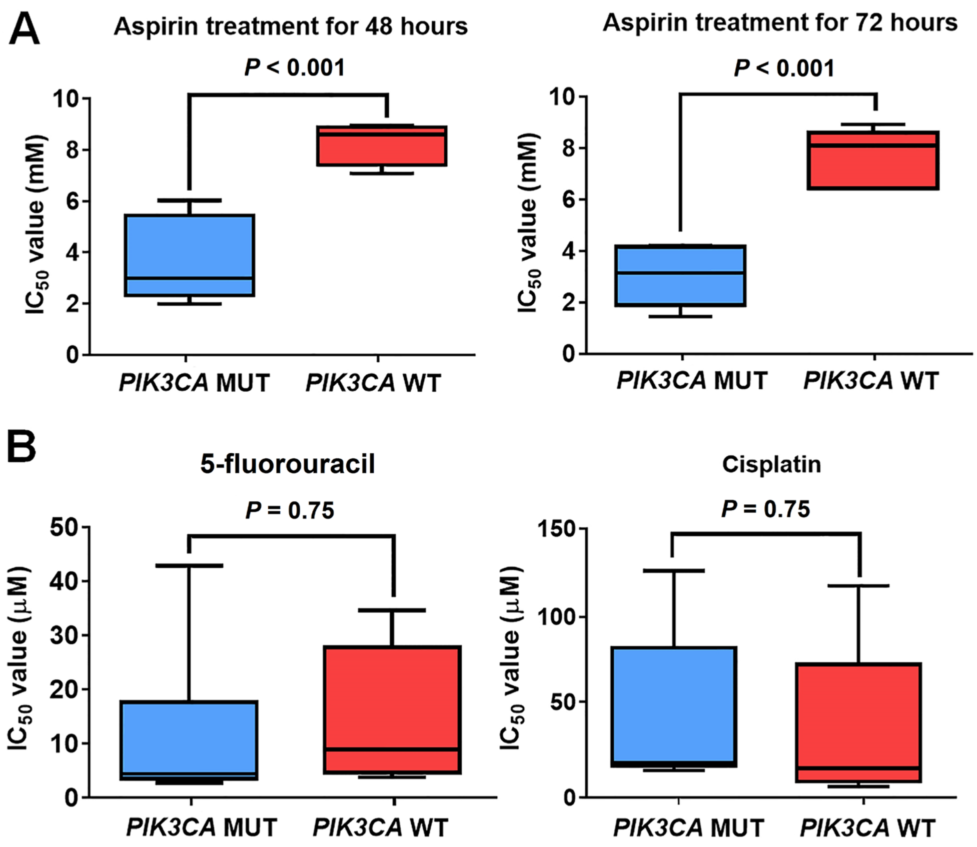

Supplementary Figure 1: IC_{50} values of aspirin and chemotherapeutic agents in colon cancer cell lines. IC_{50} values following treatment with aspirin for 48 hours (A, left) or 72 hours (A, right) in the human colon cancer cell panel were calculated using sigmoidal curve fitting. IC50 values of 5-fluorouracil (B, left) or cisplatin (B, right) in human colon cancer cell panel were quoted from the compound sensitivity database of cancer cell line encyclopedia (CCLE). Student's *t*-test was performed to determine significance between *PIK3CA*-mutant and *PIK3CA*-wild-type groups. Data represent mean ± standard deviation of three replicates. IC_{50}, half maximal inhibitory concentration; MUT, mutation; WT, wild-type.
